# Sequence and Structure Analysis of Distantly-Related Viruses Reveals Extensive Gene Transfer between Viruses and Hosts and among Viruses

**DOI:** 10.3390/v7102882

**Published:** 2015-10-19

**Authors:** Silvia Caprari, Saskia Metzler, Thomas Lengauer, Olga V. Kalinina

**Affiliations:** 1 Department for Computational Biology and Applied Algorithmics, Max Planck Institute for Informatics, Campus E1 4, 66123 Saarbrücken, Germany; scaprari@mpi-inf.mpg.de (S.C.); smetzler@mpi-inf.mpg.de (S.M.); lengauer@mpi-inf.mpg.de (T.L.); 2 Saarbrücken Graduate School of Computer Science, University of Saarland, Campus E1 3, 66123 Saarbrücken, Germany

**Keywords:** viral evolution, horizontal gene transfer, viral hallmark genes, structure comparison, hemagglutinin esterase

## Abstract

The origin and evolution of viruses is a subject of ongoing debate. In this study, we provide a full account of the evolutionary relationships between proteins of significant sequence and structural similarity found in viruses that belong to different classes according to the Baltimore classification. We show that such proteins can be found in viruses from all Baltimore classes. For protein families that include these proteins, we observe two patterns of the taxonomic spread. In the first pattern, they can be found in a large number of viruses from all implicated Baltimore classes. In the other pattern, the instances of the corresponding protein in species from each Baltimore class are restricted to a few compact clades. Proteins with the first pattern of distribution are products of so-called viral hallmark genes reported previously. Additionally, this pattern is displayed by the envelope glycoproteins from *Flaviviridae* and *Bunyaviridae* and helicases of superfamilies 1 and 2 that have homologs in cellular organisms. The second pattern can often be explained by horizontal gene transfer from the host or between viruses, an example being *Orthomyxoviridae* and *Coronaviridae* hemagglutinin esterases. Another facet of horizontal gene transfer comprises multiple independent introduction events of genes from cellular organisms into otherwise unrelated viruses.

## 1. Introduction

Unlike cellular organisms, viruses do not constitute a monophyletic group, in which the phylogenetic history can be traced back to a common ancestor. The origin and relatedness of different virus families is currently a subject of active discussion. It is unclear whether viruses have evolved by reduction of genes from cellular species, whether they descended from mobile elements of other organisms or whether they precede cellular life and are ancient self-replicating units. Possibly, all of these hypotheses are true, for a subset of viral families [1]. The recent discovery of giant viruses [2,3] revived this discussion with suggestions that a certain clade of giant viruses may represent a fourth domain of life [4].

Viruses are routinely classified according to the composition of their genome and the morphology of the viral particle, as defined by the International Committee on Virus Taxonomy [5], into families that can be grouped by the virus genome type, as represented by the Baltimore classification [6]. The genome type can be either DNA or RNA, single- or double-stranded and, in the case of a single strand, positive- or negative-sense. Retroviruses are considered a separate group that includes DNA- and RNA-encoded viruses. Altogether, this results in seven top-level classes. Lower-level groups comprise viral orders, families, sub-families, genera and species. Viruses with certain types of genome-encoding nucleic acid have been traditionally associated with different domains of life: Archaea and Bacteria usually harbor viruses with a DNA genome, whereas eukaryotes harbor RNA and retrotranscribing viruses. However, notable exceptions exist, and most viral genome types are spread across different domains. Generally, the similarity of sequences and virion structures is evident only up to the family level. In isolated cases, sequence homology can be detected between proteins of viruses with unrelated architecture, but it is unclear whether this can be attributed to horizontal gene transfer between ancestors of viral families or if this is a sign of a genuine evolutionary link, as discussed in great detail in [7,8] and other related work. It is hypothesized that in certain cases, the conserved viral genes are witnesses of evolutionary processes as ancient as the origin of life itself [7].

Another approach to the inference of viral phylogenies, capable of reaching into the distant past, is based on the comparison of the three-dimensional structures of viral capsid proteins [9]. It is well known that the three-dimensional structure of proteins is conserved over larger evolutionary distances than their sequences, and thus, several viral families can share a common fold of the capsid, although the sequences have diverged beyond similarity detection [8]. In certain cases, such viruses can be proven to have a common origin, particularly when the Baltimore class is also the same [10], but the relatedness to viruses of a different Baltimore class is more difficult to establish [11]. Therefore, an alternative hypothesis of viral evolution has been proposed that considers the capsid as the central defining element of the viral evolution, and viruses are suggested to be called “capsid-encoded organisms” [12]. A comparison of capsid proteins allowed for proposing a novel classification of viruses into so-called lineages [13]. A lineage unites several viral families, whose capsid proteins have similar folds, but otherwise, their proteins can share little or no sequence similarity. Whereas the dimensions and subunit organization of the capsids can vary within the same lineage, virions often possess other common structural features, e.g., spikes attached to five-fold icosahedral vertices of the capsid [13]. Viral lineages also may share certain details of genome organization [14].

Finally, the evolution of viruses can be traced beyond the virus world itself. Virus evolution has been linked to selfish genetic elements [15,16]. By means of the comparison of protein structures and genome organization, a subset of DNA viruses has been shown to be related to polintons, a class of eukaryotic transposons and mitochondrial plasmids [16]. However, this approach does not provide a unified picture of viral evolution due to the lack of structural data and of our ability to compare structures sharing little similarity.

Horizontal gene transfer (HGT) is one of the major forces that drive evolution. It is recognized to play a major role in the evolution of bacterial virulence and resistance [17,18]. Viruses, specifically retroviruses, facilitate horizontal gene transfer in eukaryotes [19,20]. However, it has never been systematically studied whether viruses exchange genetic information between each other via horizontal gene transfer.

There is anecdotal evidence that some viruses have experienced horizontal gene transfer. It has been proposed that viruses of the nucleocytoplasmic large DNA virus (NCLDV) group have acquired many of their genes via HGT [21]. For example, many sphingolipid biosynthesis pathway genes are shared between the microalga *Emiliania huxleyi* and its large dsDNA virus EhV-86 [22]. However, the direction of the gene transfer is unknown in this case. *Chordopoxvirinae*, a subfamily of the *Poxviridae* family, have acquired many proteins from eukaryotes, which is evidenced by their higher similarity to the eukaryotic counterparts than to other viral proteins [23]. The Sputnik phage that infects Mimivirus has borrowed a large fraction of its proteins from its host, possibly the first virus-to-virus horizontal gene transfer event documented [24]. On the other hand, this is again an event of gene capture from the host, which in this case is, quite unusually, another virus.

In this work, we have used proteins with detectable sequence or structural similarity from distant viruses, such as those with different genome types, as a starting point to investigate the evolutionary relationships among viral and cellular proteins. The major goal of the study was to detect the unlikely event of horizontal gene transfer between seemingly unrelated viruses. Through comparison of protein sequences, we have identified proteins from several protein families that appear in very distant viruses, with a conserved function, but with an origin that cannot be traced back to a single viral class, possibly pointing to several HGT events from the host. The sequence similarity in viral proteins quickly becomes undetectable with the increase of evolutionary distance. Here, we report an all-to-all comparison of the three-dimensional structures of viral proteins from different function classes that reveals many more relationships between proteins of viruses in different Baltimore classes, both structural and enzymatic. Overall, we detect two patterns in the evolution of viral proteins. Some protein families are populated by many viral proteins originating from species in different Baltimore classes and probably date back to ancient evolutionary events that led to a wide spread of certain folds in viruses. In other cases, only a small set of viruses harbor proteins similar to those from an unrelated family, in which case horizontal gene transfer events can be usually pinpointed, both between viruses and from host to virus.

## 2. Materials and Methods

Methodologically, the study consists of two parts: a comparison of protein sequences and structures. For the sequence comparison, all complete viral proteomes (as of June 2015) were downloaded from UniProt [25], and all proteins were compared pairwise with BLAST (version 2.2.21) [26]. Only hits with e-value less than 1e-05, sequence identity more than 30% and alignment length more than 50 amino acids were kept. Of these pairs, we selected those where the sequences come from viruses belonging to different Baltimore classes. The resulting sequences were scanned against the Pfam database of protein families [27] with HMMer (HH-suite Version 2.0.15) [28] using the default inclusion threshold of 0.01. Then, the corresponding HMM profiles were used to collect all related sequences from the reference viral proteome set. Maximum-likelihood phylogenetic trees were constructed with RAxML (Version 8.0.24) [29] using the PROTGAMMAJTT model and 100 replicates for the calculation of the bootstrap support. The trees were visualized with Dendroscope (Version 3.2.10) [30]. The corresponding nucleotide sequences were retrieved from ENA [31], and their GCcontent was analyzed using CodonW [32].

For the structural analysis, known three-dimensional structures of viral proteins that are the major components of the virion particle (capsid, matrix and envelope for the enveloped viruses) and structures of viral enzymes were collected from the Protein Data Bank [33] in January 2015 and grouped into different datasets depending on their biological function. The corresponding amino acid sequences were clustered using CD-HITof the CD-HIT Suite web server [34], and the structure corresponding to the longest sequence in each cluster was retained as its representative to yield a non-redundant set of protein structures with a sequence identity cut-off of 30%. This identity threshold was chosen because proteins with higher sequence identity have already been considered in the sequence analysis, so no significant hits can be lost in this way. The structural similarity between each pair of viral proteins was calculated using TMalign [35] and TM-score [36], as well as the root mean square deviation of ${\text{C}}_{\alpha }$ atoms (RMSD) were calculated for each pair of structures. TM-scores were normalized by the length of the alignment. TM-scores are designed to be comparable among the alignments of different length, and a TM-score greater than 0.5 usually means that the proteins have the same fold [37], so only these hits were retained. In contrast, RMSD depends on the length of the superimposed protein segments and thus is unreliable in large-scale comparisons. Again, we selected the pair where the proteins belong to viruses from different Baltimore classes. We performed visual inspection of the structural alignments to ensure their quality. To search for homologous structures in cellular organisms, the Protein Data Bank was scanned using the DALI [38] structure comparison server (Version 3), which provides a Z-score to measure the significance of the observed structural similarity. Structural similarities with a Z-score greater than two were considered significant. Similar structures were superimposed and displayed with UCSF Chimera (Version 1.10.2) [39].

## 3. Results

### 3.1. Sequence Analysis

All complete viral proteomes available in the UniProt database [25], comprising 32,203 sequences as of June 2015, were subjected to a pairwise comparison with BLAST [26] with additional filters to ensure the statistical and biological significance of the hits. We have considered hits only between proteins coming from viruses of different Baltimore classes, to keep only the most interesting homologs in the analysis. Significant BLAST hits that met our criteria involved all Baltimore classes (Table 1, Supplementary Table S1) and comprised 143 protein pairs of 78 different proteins.

**Table 1:** Protein families containing proteins with significant sequence similarity from viruses from different Baltimore classes.

Pfam Family	Structure	Number of	Matched Viruses and Their Types
	Representative	Similar Pairs	
	PDB ID		
*Balanced sequence distribution*			
Helicase C	4C9B	3	Molluscipoxvirus (dsDNA)
			– Pestivirus (positive-strand ssRNA)
Parvo NS1	1U0J	5	Bocavirus (ssDNA)
			– Fowl adenovirus A (dsDNA)
RdRP 1	2EC0	2	Cryspovirus (dsRNA)
			– *Potyviridae* (positive-strand ssRNA)
RNA helicase	–	4	Norovirus (positive-strand ssRNA)
			– Circovirus (ssDNA)
*Unbalanced sequence distribution*			
dUTPase	1SYL	106	Betaretrovirus (retro-transcribing ssRNA)
			– several dsDNA viruses
Hema esterase	3I27	4	Influenza C virus (negative-strand ssRNA)
			– *Coronaviridae* (positive-strand ssRNA)
HSP70	2V7Y	1	Cafeteriavirus (dsDNA)
			– Velarivirus (positive-strand ssRNA)
MMTV SAg	–	2	Betaretrovirus (retro-transcribing ssRNA)
			– Rhadinovirus (dsDNA)
OrfB IS605	–	4	Inovirus (ssDNA)
			– *Myoviridae*, Bicaudavirus (dsDNA)
Phage integrase	1AIH	4	Inovirus (ssDNA)
			– *Caudovirales* (dsDNA)
Pkinase	2IVS	5	Alpharetrovirus (retro-transcribing ssRNA)
			– Mimivirus (dsDNA)
*Not considered in detail*			
Parvo coat N	–	2	Gammabaculovirus (dsDNA)
			– *Densovirinae* (ssDNA)
–	–	1	Alphanudivirus (dsDNA)
			– Ambidensovirus (ssDNA)

For each hit pair, we searched the Pfam database [27] for the corresponding family by aligning both proteins to their HMM profiles, and in each case, both proteins turned out to belong to the same Pfam family. Hence, we refer to the hits using these family names (Table 1, Supplementary Table S1). One hit pair did not show significant similarity to any of the Pfam family. Since both proteins are annotated as hypothetical, we did not analyze this pair further. We also do not discuss the Parvo coat N family in detail, since the similarity is borderline in this case and does not allow for reliable phylogeny reconstruction.

The Pfam families are unevenly populated with pairs of similar proteins from distant viruses, the dUTPase family having the highest number of hits. We analyzed the conservation profile in all families and in the identified viral proteins. Where a three-dimensional structure of one of the proteins was available, we have mapped sequence differences onto it and analyzed their distribution with respect to active sites and other important protein regions. Next, we collected all viral sequences significantly similar to the HMM profile of the corresponding Pfam family and constructed maximum likelihood phylogenetic trees with bootstraps. The analysis of these trees reveals two distinct patterns of the distribution of viral sequences: either sequences from viruses from two Baltimore classes are present in comparable and large numbers, and the bootstrap support values do not allow for distinguishing whether the parts of the tree covered by each Baltimore class are monophyletic (balanced distribution); or one or very few sequences from one Baltimore class cluster together on a single branch of the phylogeny with a good bootstrap support, and the rest of the tree is interspersed with sequences from viruses from the other Baltimore class (unbalanced distribution). We will consider now each of these scenarios in detail and discuss the possible evolutionary implications.

#### 3.1.1. Balanced Viral Sequence Distribution

**Figure 1:**
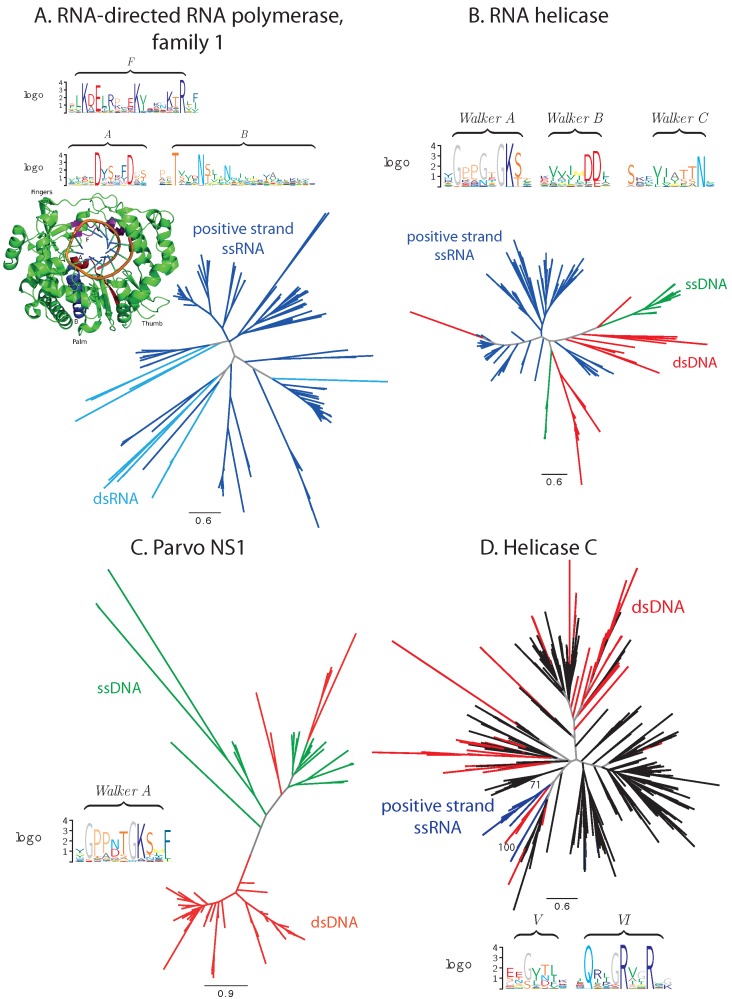
Phylogenetic and conservation analysis in families with balanced sequence distribution. (**A**) RdRP family 1; (**B**) RNA helicase family; (**C**) parvo NS1 family; (**D**) helicase C family. Branches corresponding to dsDNA viruses are colored red, ssDNA viruses green, positive-strand RNA viruses blue, ssRNA viruses cyan, proteins from cellular organisms in black and internal branches that do not lead to proteins from monophyletic clades in gray. Functional motifs and catalytic sites are presented in sequence logos and protein 3D structure, where appropriate (discussed in the text).

Several protein families exhibit a balanced distribution of the virus species from the two Baltimore classes in question. Examples include the RNA-directed RNA polymerase family 1 (PF00680, positive-strand ssRNA and dsRNA viruses), RNA helicase (PF00910, a family of the superfamily 3 helicases, positive-strand ssRNA, as well as ssDNA and dsDNA viruses), the parvo replication initiator NS1 family (PF01057, ssDNA and dsDNA viruses) and the helicase C family (PF00271, C-terminal domain of helicases from superfamilies 1 and 2, positive-strand ssRNA and dsDNA viruses) (Figure 1).

RNA-directed RNA polymerases (RdRP) and RNA helicases of superfamily 3 (S3H) are established hallmark viral proteins and were proposed to be present already in the ancient virus world [7]. RNA helicases demonstrate substantial conservation of essential functional motifs from the family of Walker motifs for NTP binding [40] and comprise viral sequences almost exclusively (Figure 1A,B). In RdRPs, the functional motifs are also well conserved. An exception is presented by RNA helicases of *Circoviridae*, where a critical lysine in motif A can be replaced by an arginine or a glutamine [41]. In the Cryspoviruses RdRPs, the functional motif F likewise contains a substitution of a lysine by a glycine, which may have an impact on the binding of the metal ions and, hence, modify the catalytic function of the enzyme [42]. However, judging from the genome composition, both proteins appear to be fully functional in their respective species. In the former case, a recombination event with a non-vertebrate-infecting virus can be suspected to have introduced this protein into vertebrate-infecting viruses [43].

The parvo NS1, also a subfamily of S3H, family consists predominantly of viral proteins, as well. Besides sequences from *Parvoviridae*, 62% of sequences identified as members of the parvo NS1 family in our analysis are E1 proteins from *Papillomaviridae*, and three and six proteins come from *Betaherpesvirinae* and *Adenoviridae*, respectively. Each of these groups is located on a separate branch with a high bootstrap support. For all of these proteins, their annotation suggests their involvement in DNA replication, and the P-loop motif characteristic for ATP/GTP binding is well conserved in the whole family (Figure 1C) [44].

Unlike the aforementioned three families that contain exclusively viral proteins, in the helicase C family, viral and cellular proteins are represented in comparable amounts (Figure 1D). Viral proteins reside on separate branches of the phylogenetic tree with a high bootstrap support. The conservation of the viral sequences is lower that of their cellular counterparts, namely of the two functional motifs [45] covered by the considered alignment; motif V (following the notation of [45]) is poorly conserved. Nevertheless, the catalytic arginines in motif VI are well conserved.

#### 3.1.2. Unbalanced Viral Sequence Distribution

**Figure 2:**
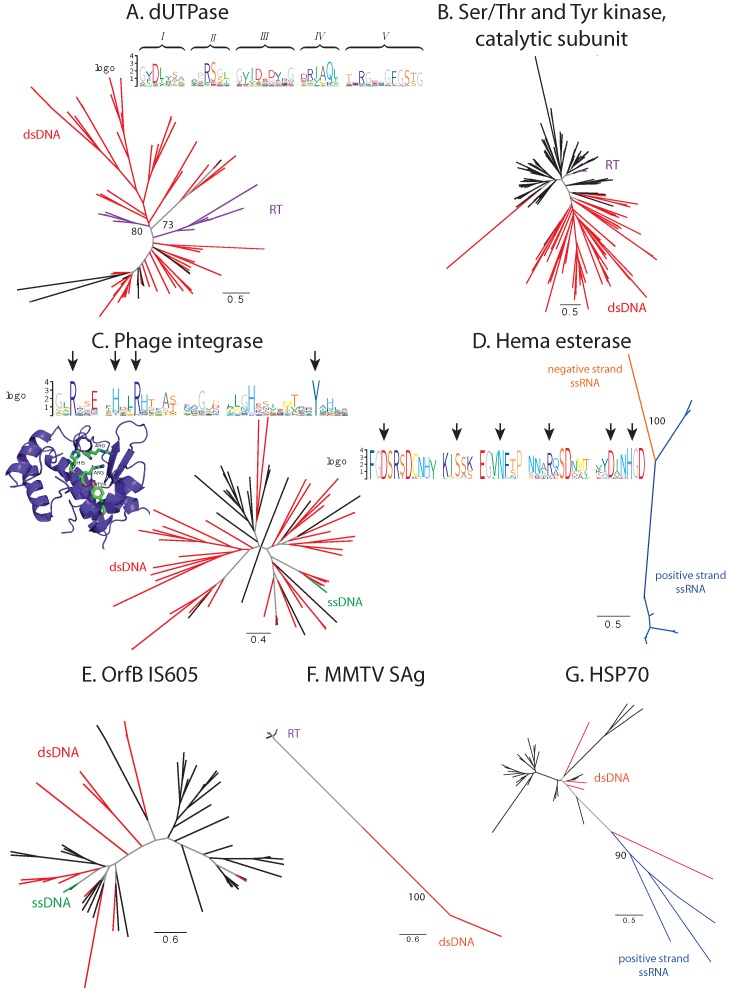
Phylogenetic and conservation analysis in families with unbalanced sequence distribution. (**A**) dUTPase family; (**B**) catalytic domain of serine/threonine and tyrosine kinase family; (**C**) phage integrase family; (**D**) Hema esterase family; (**E**) OrfB IS605 family; (**F**) MMTV SAg family; (**G**) HSP70 family. Branches corresponding to dsDNA viruses are colored red, ssDNA viruses green, positive-strand RNA viruses blue, negative-strand RNA viruses orange, retroviruses purple, proteins from cellular organisms in black and internal branches that do not lead to proteins from monophyletic clades in gray. Functional motifs and catalytic sites are presented in sequence logos and protein 3D structure, where appropriate (discussed in the text). Bootstrap support values are displayed on branches where appropriate.

A number of families demonstrate a highly unbalanced distribution of sequences between two classes of viruses: one of the classes constitutes the majority of viral proteins in the family, while the second is represented by only a few sequences. These families include: the dUTPase family (PF00692, dsDNA and retroviruses), the catalytic domain of the serine/threonine and tyrosine kinase family (PF07714, dsDNA and retroviruses), the phage integrase family (PF00589, dsDNA and ssDNA viruses), the Hema esterase family (PF03996, positive- and negative-strand ssRNA viruses), the OrfB IS605 domain family that is found in transposases (PF01385, dsDNA and ssDNA viruses) and the MMTV SAg family (dsDNA and retroviruses) (Figure 2).

dUTPases are ubiquitous in cellular organisms and catalyze the hydrolysis of dUTP to dUMP to prevent the incorporation of dUTP into DNA [46]. These proteins are also present in a large number of dsDNA viruses from *Herpesvirales*, *Poxviridae* and various bacteriophages. Each of the viral families resides on a separate branch of the phylogenetic tree; however, the bootstrap support of these branches is low. In certain cases, there is significant sequence identity between the viral protein and the dUTPase from its host, e.g., Orf virus dUTPase is 72% identical to the human dUTPase and has a GC content that is closer to that of human than of the virus itself (0.552 for the Orf virus dUTPase, 0.554 for human, 0.854 for Orf virus), suggesting recent HGT from human to virus. The bifunctional nucleocapsid-dUTPase protein from Mason-Pfizer monkey Betaretrovirus (MPMV) and protease from simian retrovirus exhibit significant sequence similarity to this family; and the MPMV protein has been proven to be an active dUTPase [47]. Phylogenetically, they are distinct from other viral dUTPases with a high bootstrap support and are most closely related to endogenous retroviruses from placental animals (Figure 2A). The five functional motifs described for dUTPases [46] are well conserved in the viral proteins, which suggests the enzymatic function to be conserved. Thus, we can reasonably assume that dUTPases of dsDNA and retroviruses were obtained through independent evolutionary events, but were recruited to serve the same function in different viral groups.

The catalytic domain of serine/threonine and tyrosine kinase is a part of kinases that can be found in all domains of life [48]. Many large eukaryotic dsDNA viruses, including the NCLDV group, contain a kinase with this domain. Additionally, it can also be found in the Rous sarcoma virus (*Alpharetrovirus*) protein Src. The proteins closest to Src are the eukaryotic kinases Fyn and Fgr (Figure 2B). Although the GC content statistics do not support this hypothesis, it is plausible that Src was transferred into the Rous sarcoma virus from its host.

The phage integrase from the single-strand DNA Pseudomonas phage Pf1 is the only representative from ssDNA viruses in this family, which consists mainly of proteins from different dsDNA bacteriophages (Figure 2C). The key catalytic residues characteristic of phage integrases [49] are conserved in this protein (marked with arrows in Figure 2C). Thus, one can conclude that the enzymatic activity has been preserved. It is plausible that the introduction of this protein into Pseudomonas phage Pf1 is due to the extensive HGT that takes place in bacteria.

The esterase domain forms a part of hemagglutinin from influenza C virus (negative-strand ssRNA) and is significantly similar to hemagglutinin-esterases from Torovirus and Betacoronavirus (positive-strand ssRNA), and all three viral species form separate clades with high bootstrap support (Figure 2D). The key catalytic residues ([50], marked with arrows in Figure 2D) are highly conserved. Thus, the proteins are probably functionally competent. The three proteins are equidistant with about 30% sequence identity. Thus, the family may have originated in any of the respective species and then spread into the others via HGT [50], although the routes of the transfer are difficult to discern. The comparison of three-dimensional structures of influenza and Betacoronavirus hemagglutinin esterases confirms that the transfer occurred from *Orthomyxoviridae* into *Coronaviridae*, and not *vice versa* (see Section 3.2.2).

The C-terminal transposase domain OrfB IS605 is found in bacteria and in a variety of dsDNA bacteriophages, as well as in a single ssDNA Enterobacteria phage If1 (Figure 2E). It is 99% identical to the *Escherichia coli* transposase, and its GC content is more similar to that of *E. coli* than to that of the phage itself (0.507 for the protein, 0.532 for *E. coli*, 0.360 for the phage). Thus, we can reasonably assume that this domain was introduced into Enterobacteria phage If1 from its host.

The MMTV SAg family is a small protein family consisting of the mouse mammary tumor virus superantigen, several endogenous copies in the mouse genome and, surprisingly, two proteins from the *Herpesrvirus* genus: immediate-early protein IE-G from saimiriine herpesvirus 2 and mitogen from ateline herpesvirus 3, which are quite distant and separated with a good bootstrap support from the rest of the family (Figure 2F). Superantigens of the MMTV SAg family cause nonspecific activation of T-cells [51]. The functional characterization of immediate-early protein IE-G from saimiriine herpesvirus indicates that its activity, boosting the T-cell proliferation upon infection [52], relates it to superantigens of the MMTV SAg family.

The HSP70 family of chaperones comprises one of the key components of the heat shock system ubiquitous in cellular organisms. Here, we identify HSP70 homologs in several giant viruses and in four members of the *Closteroviridae* family (Figure 2G). While preserving their function in protein folding in the giant viruses [2], these proteins have acquired additional functions in closteroviruses as tail integral components [53].

We have also identified weak similarity between an uncharacterized protein from Neodiprion lecontei nucleopolyhedrovirus and several Parvovirus coat proteins. Although horizontal gene transfer of a different gene, NS3, has been suggested before between *Parvoviridae* and *Baculoviridae* [54], the similarity of coat proteins is borderline and does not allow for definitive conclusions.

Additionally, we have performed a more sensitive search among all viral proteins with HMMer [28] using all Pfam HMMs (Supplementary Table S2). We have identified 28 additional families that have their representatives in viruses from different Baltimore classes and observe a diverse distribution of hits among diverse Baltimore classes (Supplementary Figure S1, Supplementary Table S3). In a few cases, possible horizontal gene transfer (baculovirus glycoprotein GP64 in Thogotovirus [55] or infectious salmon anemia virus-like hemagglutinin in anguillid herpesvirus 1 [56]) or common evolution (movement protein family MP [57]) has previously been discussed. In others, nothing is known (e.g., we have identified the presence of a Corona_NS2 domain in VP3 of rotavirus with a significant e-value of 2.7e-06 for the first time), but the weak sequence similarity does not allow for suggesting a definitive evolutionary scenario.

### 3.2. Structural Analysis

To investigate the relatedness of proteins, whose similarity can be detected at the level of protein structures, but not sequences, we have performed structural comparison. The structures of the capsid, matrix and envelope proteins and of the viral enzymes were collected from Protein Data Bank [33] in January 2015 and grouped into different datasets according to their biological function, based on their annotation. For each dataset of proteins, sequences with greater than 30% sequence identity were clustered together, since such proteins are almost always homologous and, hence, have very similar three-dimensional structures [58]. We compared the three-dimensional structures of proteins representing each cluster and calculated RMSD and TM-scores [35] for structural alignments. Although intuitive, RMSD is a poor measure for comparing structural similarity across different protein folds: it depends on the length of the aligned regions, in that small protein segments tend to be aligned with smaller RMSD than large and complex structures. Nevertheless, the structural similarity of large protein fragments bears more evolutionary information. TM-score, in contrast, is devoid of these weaknesses and was normalized by the alignment length. It has been shown that TM-scores greater than 0.5 almost always correspond to the alignment of structures with a similar fold [37].

As phylogenetic reconstruction is hardly applicable in the absence of detectable sequence similarity, we did not perform it in this part and discuss mere protein distribution within groups with similar three-dimensional structure. To do so, we searched the Protein Data Bank for the structures of proteins from cellular organisms similar to the viral proteins from each considered functional class. We have been able to identify several folds observed exclusively in viruses and others with structural homologs in cellular species. The virus-specific folds identified here complete the list of the hallmark viral proteins [7].

We have analyzed 696 three-dimensional structures from the following functional classes: capsid, envelope, esterase, glycosyltransferase, helicase, kinase, ligase, lyase, matrix, methyltransferase, nuclease, oxidoreductase, polymerase, protease and terminase. For two classes of structural proteins and four classes of enzymes, we observe structural similarities between proteins of viruses from different Baltimore classes (Table 2, Supplementary Table S4). Analyzing the spread of the respective proteins in the corresponding viral families, one can again observe two patterns: some proteins can be found in many viral families and ubiquitous within these families, and others are found in typically two families from different Baltimore classes and even within them are confined to a few genera. In the absence of the possibility of conducting a proper evolutionary analysis of the respective protein clusters due to the virtual lack of sequence similarity, we refrain here from calling them “families” and refer to these two groups as widely populated and confined folds, respectively.

**Figure 3:**
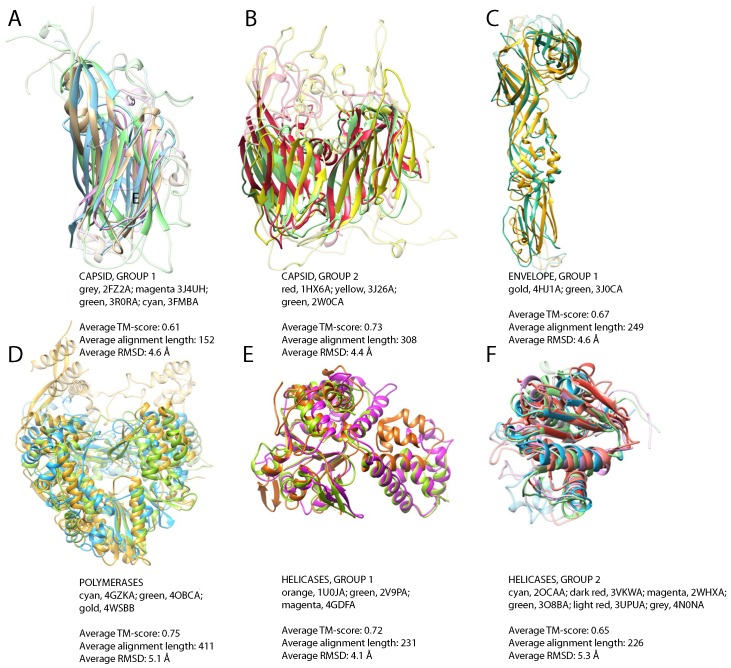
Widely-populated folds, superposition of representative structures. (**A**) Jelly-roll fold domains of Turnip yellow mosaic virus, positive-strand ssRNA genome (grey), Bordetella phage BPP-1, dsDNA (magenta), porcine circovirus-2, ssDNA (green) and infectious bursal disease virus, dsRNA (cyan); (**B**) double jelly-roll fold motifs of major capsid protein from Enterobacteria phage PRD1, dsDNA (red), V20 of Sputnik, unclassified virophage (yellow) and major capsid protein P2 of Pseudoalteromonas phage PM2, dsDNA (green); (**C**) superposition of envelope glycoproteins from Rift Valley fever virus, negative-strand ssRNA (gold), Venezuelan equine encephalitis virus, strain TC-83, positive-strand ssRNA (green); (**D**) RdRP of Pseudomonas phage phi12, dsRNA (cyan), hepatitis C virus JFH-1, positive-strand ssRNA (green), influenza A virus, strain A/little yellow-shouldered bat/Guatemala/060/2010 (H17N10), negative-strand ssRNA (gold); (**E**) helicase domains of DNA replication protein from adeno-associated virus 2, ssDNA (orange), the replication protein E1 from bovine papillomavirus type 1, dsDNA (green), large T antigen of simian virus 40, dsDNA (magenta); (**F**) RecA-like domains of ATP-dependent DNA helicase uvsW from Enterobacteria phage T4, dsDNA (cyan), replicase large subunit from tobacco mosaic virus strain tomato/L, positive-strand ssRNA (dark red), NS3 helicase from Dengue virus 4, positive-strand ssRNA (magenta), NS3 helicase from hepatitis C virus subtype 1b, positive-strand ssRNA (green), ATP-dependent DNA helicase dda from Enterobacteria phage T4, dsDNA, (light red), replicase polyprotein 1ab from equine arteritis virus Bucyrus, positive-strand ssRNA, (grey). For clarity, non-superimposable loops were dimmed.

#### 3.2.1. Widely-Populated Folds

In the non-redundant set of 132 structures of capsid proteins, we detect two large groups of similar structures (Table 2, Supplementary Table S4). The first group comprises protein chains of 50 known structures with an average TM-score of 0.61, encompassing proteins from positive-strand ssRNA, ssDNA, dsDNA and dsRNA viruses. All of them share the presence of the jelly-roll fold (Figure 3A). This structural motif has been previously reported to be found in a wide variety of viruses, including ssRNA, ssDNA, dsDNA and dsRNA viruses [8,59]. There are no structural homologs of the jelly-roll capsids among the proteins from cellular organisms, and jelly-roll capsids also belong to the viral hallmark proteins [7].

**Table 2:** Widely-populated and confined folds. For each dataset, clusters of similar viral structures with the respective PDB identification codes (three-character code and one-letter chain identifier) of the subunits considered are reported. For each cluster, a brief description of the biological function of the proteins is also given, as well as information about the type of genome of viruses falling in the same cluster.

Dataset	Groups	PDB ID and Chain	Description	Classes of Viral Genomes
*Widely Populated Folds*				
Capsid	Group 1	3NAPB, 1B35B, 4QPIB,	Capsid subunits with jelly roll fold	positive-strand ssRNA, ssDNA, dsDNA, dsRNA
		3CJIC, 4GMP0, 3CJIB,		
		1BEV3, 4QPIC, 1B35C,		
		3NAPC, 1F2NA, 1X35A,		
		2IZWA, 2FZ2A, 3ZXAC,		
		1OPOA, 2TBVA, 2WS91,		
		3CJIA, 4Q4W1, 4QPIA,		
		2MEV1, 1B35A, 3NAPA,		
		3IYOA, 2GH8A, 4NWVA,		
		1IHMA, 3J1PA, 1CWPA,		
		1F15A, 4V4MA, 3J4UH,		
		3J40H, 1YQ5A, 1A6CA,		
		1S58A, 3J1QA, 4G0RA,		
		3N7XA, 1DNVA, 3P0SA,		
		3R0RA, 2BBVA, 2WUYA,		
		2W0CL, 1SVA1, 1DZLA,		
		3FBMA, 1OHFA		
	Group 2	1HX6A, 2BBDA, 2W0CA,	Capsid subunits with double jelly roll fold	dsDNA, unclassified virophage (Sputnik)
		1M3YA, 3J26A		
Envelope	Group 1	4HJ1A, 3J0CA, 4ADIA,	Envelope glycoproteins	positive-strand ssRNA,
		3J27A, 2GG1A		negative-strand ssRNA
Poly-		4GZKA, 4A8OA, 2R7RA,	RNA-dependent	dsRNA,
merases		1N35A, 3ZEDA, 4WSBB,	RNA polymerases	positive-strand ssRNA
		4OBCA, 2CJQA, 4HDHA,		negative-strand ssRNA
		2EC0A, 3UQSA, 1KHVA		
Helicases	Group 1	2V9PA, 4GDFA, 1U0JA	Superfamily 3 helicases	dsDNA, ssDNA
	Group 2	3VKWA, 4N0NA, 3UPUA,	Superfamily 1 and 2 helicases	positive-strand ssRNA, dsDNA
		2OCAA, 3O8BA, 2WHXA		
*Confined folds*				
Proteases		4QBBA, 4M0WA, 2J7QA,	papain-like	positive-strand ssRNA,
		4IUMA, 3MTVA	cysteine proteases	dsDNA
Methyl-		3MAGA, 2XYQA, 3EMDA	Methyltransferases	dsDNA
transferases				positive-strand ssRNA
Envelope	Group 2	2WR1A, 3BT6A, 1FLCA,	Hemagglutinin,	negative-strand ssRNA,
		3CL5A	Hemagglutinin esterases	positive-strand ssRNA

The second group represents capsid proteins from viruses with dsDNA genome (Enterobacteria phage PRD1, Sulfolobus turreted icosahedral virus 1, Pseudoalteromonas phage PM2 and Paramecium bursaria Chlorella virus 1) and the structure of the capsid protein V20, belonging to the unclassified virophage Sputnik [60] (Table 2, Figure 3B). The Sputnik virophage is from the same Baltimore class, dsDNA, as its host, Mimivirus, and excessive HGT from the host has been reported in this case [24]. Therefore, although these viruses represent distant families, all of them have the same type of genome. The fold in these structures has been dubbed double jelly roll, and it is also a frequent motif in viral capsid proteins [61,62,63,64]. As is typical for capsid proteins, this fold is also found exclusively in viruses.

The 43 envelope proteins also comprise two structural groups (Table 2). The first one consists of glycoproteins of negative-strand ssRNA viruses (Rift Valley fever virus) and positive-strand ssRNA viruses (Venezuelan equine encephalitis virus strain TC-83, Rubella virus, Dengue virus 2, Langat virus), collectively called class II fusion proteins. The average TM-score within positive-strand ssRNA viruses is similar to that between negative-strand ssRNA and positive-strand ssRNA viruses and is 0.67. The structural similarities (Figure 3C) detected among these proteins are in agreement with the similarities previously reported between flaviviruses and alphaviruses and the proposed evolutionary link between *Bunyaviridae* and *Flaviviridae* [65]. This fold is structurally similar to cell-cell fusion protein EFF-1 from *C. elegans*, although sequence homology is not detectable (DALI Z-scores between 4.9 and 12.3, RMSD between 2.9 Å and 12.1 Å, sequence identity between 5% and 9%, Supplementary Figure S2). The analogy of the fusion mechanism between the viral and eukaryotic proteins has been suggested [66]. The second structural group among envelope proteins represents an example of HGT and will be discussed in the subsequent section.

In the set of 28 polymerases, we detected significant structural similarities among dsRNA viruses (Pseudomonas phage phi12, Pseudomonas phage phi6, simian rotavirus, mammalian orthoreovirus 3 Dearing, infectious pancreatic necrosis virus), negative-strand ssRNA viruses (influenza A virus, strain A/little yellow-shouldered bat/Guatemala/060/2010 H17N10) and positive-strand ssRNA viruses (hepatitis C virus JFH-1, bovine viral diarrhea virus 1, Japanese encephalitis virus, foot and mouth disease virus C-S8c1, murine norovirus 1, rabbit hemorrhagic disease virus) (Table 2 and Figure 3D). All of these enzymes have an RNA-directed RNA polymerase (RdRP) activity. Thus, we extend the set of related RdRPs identified by sequence comparison. No structural homologs of RdRPs have been found among the proteins from cellular organisms, in agreement with their unique function in RNA viruses. Here, we show that sequence-structure homology spans all classes of RNA viruses. RdRPs from dsRNA and positive-strand ssRNA viruses have previously been reported to be related [67]. Now with resolved three-dimensional structures of RdRPs from negative-strand ssRNA viruses emerging [68], one can argue that RdRPs from RNA viruses share a common fold. This agrees with an earlier hypothesis that negative-strand ssRNA RdRPs evolved from positive-strand ssRNA virus RdRPs [7]. RdRPs from Eukaryota have been reported, but they are structurally distinct, showing more similarity to DNA-dependent RNA polymerases [69].

As noted above, the superfamily 3 helicase family is one of the viral hallmark protein families [7], and similarity between these helicases in viruses with different genome types can be detected already at the sequence level. At the structural level, we detect the similarity between the structures of the E1 hexameric helicase of the bovine Papillomavirus type 1, of the simian virus 40 initiator/helicase (both dsDNA) and of the DNA replication protein belonging to adeno-associated virus-2 (ssDNA) (Table 2 and Figure 3E).

For other helicase superfamilies, we detected a group of structurally-similar superfamily 1 helicases (S1H) containing proteins from viruses with positive-strand ssRNA genome (tomato mosaic virus strain tomato/L and equine arteritis virus strain Bucyrus) and with dsDNA genome (Enterobacteria phage T4) and of structures of the superfamily 2 helicases (S2H) containing proteins from viruses with dsDNA genome (Enterobacteria phage T4) and two structures of HCV NS3 protease/helicase proteins belonging to positive-strand ssRNA viruses (hepatitis C virus subtype 1b and Dengue virus 4). All of these structures can be readily superposed using their C-terminal RecA-like domain (Table 2 and Figure 3F), a motif which has an architecture shared by helicases S1H and S2H [70]. The grouping of S1H and S2H helicases into a common cluster is in agreement with them putatively sharing a common ancestor [71]. This extends the observed sequence similarity in the helicase C family.

Both groups of helicases also have structural homologs outside the viral world. Viral helicases of superfamily 3 have significant similarity to several human proteins, in particular the human AAA+ ATPase Tip49b (DALI Z-scores between 7.3 and 8.5, RMSD between 3.2 Å and 3.7 Å; Supplementary Figure S3). The function of these proteins as ATP-dependent hydrolases is also similar. Superfamily 1 and 2 helicases are also known to be conserved in cellular organisms, including bacteria and humans [70].

#### 3.2.2. Confined Folds

**Figure 4:**
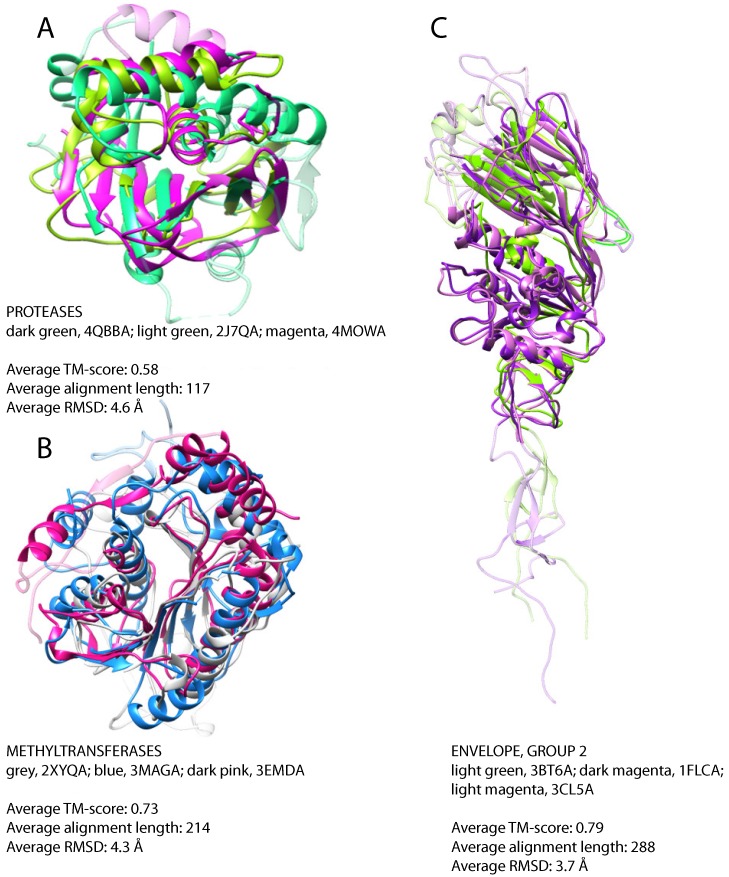
Confined folds, superposition of representative structures. (**A**) Protease domains of leader protease from foot and mouth disease virus, strain O1, positive-strand ssRNA (dark green), ubiquitin specific protease from murine herpesvirus 1, dsDNA (light green), papain-like protease from SARS coronavirus, positive-strand ssRNA (magenta); (**B**) mRNA cap-specific methyltransferase VP39 from Vaccinia virus, dsDNA (blue), putative 2’-O-methyl transferase from SARS coronavirus, positive-strand ssRNA (grey), methyltransferase from the Wesselsbron virus, positive-strand ssRNA (dark pink); (**C**) hemagglutinin-esterase-fusion glycoproteins of influenza C virus, strain C/Johannesburg/1/66, negative-strand ssRNA (dark magenta), influenza B virus, strain B/Memphis/13/2003, negative-strand ssRNA, (light green), bovine coronavirus, positive-strand ssRNA (light magenta). For clarity, non-superimposable loops were dimmed.

In the set of 36 protease structures, we detect similarity between cysteine proteases with a papain-like fold belonging to viruses with positive-strand ssRNA and dsDNA viruses (Table 2 and Figure 4A). In particular, M48, ubiquitin-specific protease of murine herpesvirus 1 that has a dsDNA genome, shows similarity with proteases of viruses with positive-strand ssRNA, e.g., the papain-like protease of SARS coronavirus, the leader protease of the foot and mouth disease virus, strain O1 (*Aphthovirus*), the papain-like protease 2 of equine arteritis virus and the papain-like cysteine protease of porcine reproductive and respiratory syndrome virus. Sequence similarity searches deliver no clues about the evolutionary history of these proteases. Indeed, homologs of the protease of the SARS coronavirus can be found in a variety of beta- and gamma-coronaviruses, while the homologs of leader protease of the foot and mouth disease virus can be found only within the genus of *Aphthovirus* and of the ubiquitin-specific protease of murine herpesvirus 1 in a small set of cytomegaloviruses. Despite the lack of sequence similarity, these proteins share the same papain-like fold. A search for structural similarities with DALI [38] revealed the presence of structural homologs in both eukaryotic and prokaryotic organisms that suggests gene transfer events (Supplementary Figure S4). The fact that each viral protease is more similar to proteases from vertebrates than to other viral proteases might suggest multiple introduction events from the host. Nevertheless, the existence of considerable tertiary structural similarity between different papain-like proteases, whose sequence similarity is not detectable, might point to structural convergence events. The low sequence similarity, non-conserved gene context and the absence of a reliable evolutionary model precludes us from suggesting a definitive evolutionary scenario. Further investigations will be necessary to elucidate the origin and evolution of proteases with a papain-like fold in viruses.

From 12 methyltransferases, we detect structural similarity between mRNA cap methyltransferases of viruses with dsDNA genome (Vaccinia virus) and positive-strand ssRNA genome (SARS coronavirus and Wesselsbron virus) (Table 2). The superposition of these three structures reveals significant structural similarity, especially between the methyltransferases of the SARS coronavirus and the of Vaccinia virus that have different genomes (Table 2 and Figure 4B). A cellular homolog of the methyltransferase from SARS coronavirus can be found in *E. coli* with 65% sequence identity and an e-value of 1e-138, and viral homologs are detected in numerous coronaviruses. The homologs of methyltransferase from Vaccinia virus can be found in several other poxviruses, while the homologs of the Wesselsbron virus are restricted to flaviviruses. However, no sequence homology between these three groups is detectable. On the other hand, structural similarities can be observed between these enzymes and methyltransferases of prokaryotic and eukaryotic organisms and may suggest gene transfer events (Supplementary Figure S5). Again, the viral proteins are more similar to eukaryotic methyltransferases than to each other, which suggests multiple introduction events from the host. The host range for all three viruses coincides with the species of the closest non-viral structural relative. Similarly to what was previously suggested for the evolution of viral papain-like proteases, we cannot exclude that the structural similarity observed between methyltransferases of different viruses and viral and some cellular methyltransferases is the result of convergent evolution events.

**Figure 5:**
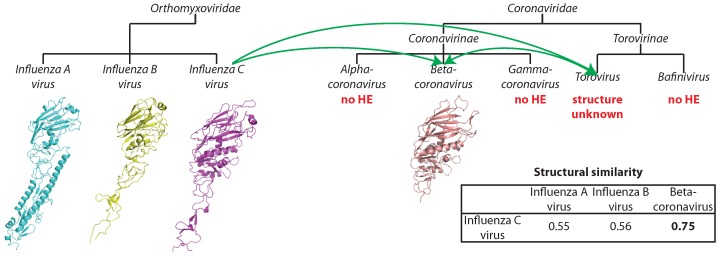
Potential route of horizontal gene transfer of hemagglutinin-esterase (HE) from *Orthomyxoviridae* to *Coronaviridae*. Structural similarity is reported as TM-score.

The second group of structurally-similar envelope proteins comprises glycoproteins belonging to negative-strand ssRNA viruses (Asian influenza, a subtype of influenza A, influenza B virus strain B/Memphis/13/03, influenza C virus strain C/Johannesburg/1/66) and to betacoronavirus of the Coronaviridae family with positive-strand ssRNA genome (Table 2 and Figure 4C). The Asian influenza and the influenza B virus glycoproteins are hemagglutinins, proteins mediating the viral fusion with the host cell membrane, while the glycoproteins of influenza C and that of betacoronavirus are hemagglutinins with an additional esterase domain (hemagglutinin esterases). Similarity between hemagglutinin esterases of betacoronavirus and influenza C virus is detectable already on the sequence level, as discussed above, but does not allow for resolving the direction of the gene transfer. Additionally, hemagglutinin esterase of Torovirus (also from *Coronaviridae*), whose structure is unknown, is similar to these two with sequence identity of about 30%. Structural similarities were previously reported between hemagglutinins of influenza A, B and C in the HA2/HEF2 chain. Similarity between the hemagglutinin esterases of coronaviruses and orthomixoviruses has been reported to be a result of lateral gene transfer events [72]. However, the direction of the transfer has not been analyzed in the original report. Taking all of the data together, we can propose that the transfer happened from influenza C virus after its split from influenza A and B around 8000 years ago [73], either to one of the Coronaviridae genera and then from there to the other or to both of them independently (Figure 5). Since the hemagglutinin esterases of betacoronavirus and Torovirus are more similar to each other than to the respective spike glycoproteins, introduction into a common ancestor of *Coronaviridae* and subsequent loss in all other genera is unlikely.

## 4. Discussion

We have performed a comprehensive analysis of similarities between viral proteins from different Baltimore classes, both at the level of sequence and of structural similarity. It is generally assumed that viruses are polyphyletic. Nevertheless, some key viral proteins, such as RNA-dependent RNA polymerases, appear to be related in very large groups of viruses, possibly all RNA viruses [7]. It has even been proposed that all positive-strand ssRNA viruses and some dsRNA viruses have a common origin [74]. Another line of evidence shows that some dsRNA, positive-strand ssRNA and dsDNA viral families share a common capsid structure [75]. The concept of viral hallmark genes has been proposed [7] to denote genes, and consequently proteins, that are shared by many diverse groups of viruses, have only very distant homologs in cellular organisms, are monophyletic within each group of genes and thus can be used to define an entity as a virus. However, these insights are related to only a few, although very important, viral proteins and are largely achieved through careful manual analysis. In this work, we have developed a procedure that requires minimal manual intervention to explore all possible candidate proteins that are common in at least two different Baltimore classes and thus are either viral hallmarks or have some other interesting evolutionary history. The fact that we recapitulate all known viral hallmark genes that can be found in more than one Baltimore class proves the validity of our approach. Some of the viral hallmark genes have been discovered only by comparing protein structures, which stresses the necessity of this type of analysis.

Our procedure involves both sequence and structural comparison. Both parts have their limitations: on the one hand, only a fraction of viral proteins have a resolved three-dimensional structure; on the other hand, comparing protein structures allows for discovering remote homologies unrecognizable by sequence comparison. Naturally, the two parts complement each other in the analysis of certain proteins (Table 3). A sequence comparison identifies all known members of each protein family, whereas structural comparison provides information on distant homologs and sometimes allows one to resolve evolutionary events. It must be noted that the number of resolved three-dimensional structures of viral proteins lags far behind the number of available sequences of them, and in the light of the new developments in the field of next-generation sequencing, this gap can be expected to be growing. Nevertheless, the information of the three-dimensional structures is essential to resolve distant homologies, as exemplified by RNA-dependent RNA polymerases.

**Table 3:** Overlapping protein families and folds identified in the sequence and structure comparison, respectively. The number of proteins from each Baltimore class is given in parentheses.

Baltimore Classes	Family		Fold	Baltimore Classes
*Families with balanced sequence distribution/widely-populated folds*				
dsRNA (22)	RdRP 1	↔	Polymerases	dsRNA (5)
positive-strand ssRNA (205)				positive-strand ssRNA (6)
				negative-strand ssRNA (1)
dsDNA (31)	RNA helicase	↔	Helicases	dsDNA (2)
ssDNA (24)			Group 1	ssDNA (1)
positive-strand ssRNA (124)				
dsDNA (101)	Helicase C	↔	Helicases	dsDNA (2)
positive-strand ssRNA (44)			Group 2	positive-strand ssRNA (4)
dsDNA (76)	Parvo NS1			
ssDNA (34)				
*Families with unbalanced sequence distribution / confined folds*				
positive-strand ssRNA (16)	Hema esterase	↔	Envelope	positive-strand ssRNA (1)
negative-strand ssRNA (3)			Group 2	negative-strand ssRNA (3)

Functionally, a unifying theme is that almost all of the proteins, the similarity of which can be detected from sequence comparison, are related to nucleic acid processing or modification: dUTPase, transposase, helicase and RNA polymerase. Comparison on the structural level adds viral structural proteins (capsid, envelope) and some protein- and RNA-modifying enzymes (proteases, methyltransferases) to this pool. Thus, we observe a network of evolutionary links between the proteins that perform very basic biochemical and structural functions in different virus families.

Studying relationships among proteins found in viruses of different Baltimore classes that nevertheless exhibit significant sequence or structural similarity, we observe two distinct patterns. Either both virus classes are represented by a large number of proteins, and the evolutionary relationships between the viral proteins (and their cellular relatives, that are present in the family) are ancient and difficult to resolve; or one of the two or both groups of proteins are present only in a few genera, often one group being underrepresented relative to the other. In the latter case, HGT can often be proposed. When comparing protein sequences, we call the first pattern “balanced sequence distribution” and the second pattern “unbalanced sequence distribution”. Since the sequence homology is in all cases evident, we can be sure that we deal with a monophyletic protein family and, hence, use phylogenetic analysis and discuss the evolutionary events that happened in that family. In the comparison of protein structures, we set the algorithm parameters such that we discover proteins that have the same fold according to the CATH [76] classification or the same fold in SCOP [77]. The common ancestry of such proteins is likely, but not necessary, so we refrain from calling these grouping protein families. Instead, we use terms “widely represented folds” and “confined fold” for the two patterns mentioned above. It must be noted that beside proteins with homologs in cellular species, all other families with balanced sequence distributions and widely represented folds correspond to viral hallmark genes. For families with unbalanced sequence distribution and confined folds, we can propose a scenario involving horizontal gene transfer (HGT) in most cases.

Using the comparison of protein three-dimensional structures, we detected similarities between viral capsids of the jelly-roll fold that are characteristic to a variety of species and also were reported among viral hallmark proteins [7]. The double jelly-roll fold is also wide-spread, but so far, has been detected in only dsDNA viruses, and the Sputnik virophage that lacked the official Baltimore classification at the moment of this writing, but is known to possess dsDNA genome [24]. Using structural comparison, we supplemented the set of superfamily 3 helicases (also a virus hallmark proteins) with another distantly-related viral representative and found a link between superfamily 1 and superfamily 2 helicases.

We have found several cases in which the structural similarity is limited to a few species and sheds light on the evolutionary relationships that otherwise are difficult to interpret due to the lack of traceable sequence similarity. In the case of hemagglutinin esterases of coronaviruses and orthomixoviruses, lateral gene transfer, but not its direction, has been previously reported [72]. We can now identify this direction as coming from the latter into the former. For cysteine proteases of Aphthovirus, coronaviruses and cytomegaloviruses and for methyltransferases of *Flaviviridae* and *Poxviridae*, the available data are insufficient to draw any concrete conclusions, but their structural similarity to proteases and methyltransferases of cellular organisms suggests that genetic transfer events happened very anciently. However, for both proteases and methyltransferases, we cannot rule out that the similarities observed with proteins of cellular organisms are the result of convergent evolution processes that caused the same fold to be present in viral and cellular proteins. Further investigations will be needed in order to elucidate the origin and evolution of these two classes of viral proteins.

MMTV superantigen (SAg) family proteins are specific to very particular viruses. We observe these proteins in unrelated virus families, where the proteins exhibit significant sequence similarity and conserved structure and function and, hence, very likely share the same evolutionary origin. Gene transfer from a common (possibly past) host is not likely, since these protein families are not represented in cellular organisms. Options for how these genes could have been acquired include gene exchange between viruses, e.g., through co-infection of the same host and recombination, or convergent evolution.

An interesting pattern of genetic exchange arises in bacteriophages. Bacteria have already been observed to donate their genes to phages [78], which leads to accelerated evolutionary rates in the latter. It is also noted that massive HGT between bacteria and their phages is likely to be a common phenomenon [78]. Here, we observe that phages co-infecting the same or related hosts can exchange genetic information (e.g., phage integrase family).

HGT can be detected in protein families with a variety of methods, which can be divided into two groups. The first group of methods is based on the analysis of a reliable phylogenetic tree of the protein family: any event in this tree that contradicts the species tree is a candidate for HGT [79]. The second group of methods relies on the analysis of the sequence statistics and special features that can be evidence for HGT if not typical for the considered species [80,81,82]. The simplest, but practical statistical measure is the GC content of the corresponding nucleotide segment. Application of these different tools to the identification of HGT events in viruses encounters difficulties, both rooted in the methodology and caused by the biology of the subject.

The elevated rates of evolution, especially in RNA viruses, could be a reason why HGT events are so difficult to detect in viruses. In the absence of a common evolutionary tree for viruses beyond their immediate families, phylogeny-based methods for HGT detection are inapplicable. Sequence statistics also does not offer a reliable tool, since the transferred segments can adjust very quickly to their new background. In fact, the only case, when the GC content analysis reliably supports HGT, involves DNA phages (OrfB IS 605 family) or dsDNA poxviruses (dUTPase of the Orf virus). Given the indications that HGT in viruses is quite common, at least in some clades, such as NCLDV [21,83], there is a great need to develop alternative strategies that do not rely on sequence comparison. In the presented work, we investigate all cases available to us of significant sequence and structural homology.

## 5. Conclusions

The extension of sequence comparison analysis at the structural level elucidates the same pattern of genetic exchange as those revealed by sequence analysis: probable acquisition from a common ancestor, potential lateral transfer from other viruses and multiple introductions from the host. Additionally, structural analysis led to new findings, e.g., it allowed us to identify an interesting example of envelope glycoproteins that are related to a cell-cell fusion protein and also resolve the evolutionary history of hemagglutinin-esterase introduction into *Coronaviridae*. These few examples hint at the potential multitude and scale of these events, which can be only fully appreciated if more data on three-dimensional structure of viral proteins become available, which may paint a much more complex picture of viral evolution than previously expected.

## Supplementary Materials

**Figure S1.** The distribution of proteins from viruses with different genome types (A) and the number of hits within the viral reference proteome (B) of protein families identified in the HMMer [28] search; **Figure S2.** Structural superposition between the envelope glycoprotein of Rift Valley fever virus (4HJ1A, cyan) and EFF-1 of *Caenorhabditis elegans* (4OJDH, orange); **Figure S3.** Structural superposition between the adeno-associated virus-2 helicase (1U0JA, yellow) and the human Tip49b ATPase (3UK6E, light blue); **Figure S4.** Structural superposition between the papain-like protease of SARS coronavirus, positive-strand ssRNA (4M0WA, cyan) and the human ubiquitin carboxyl-terminal hydrolase 21 (2Y5BA, green); **Figure S5.** Structural superposition between VP39 of Vaccinia virus, dsDNA (3MAGA, dark red) and human catechol-O-methyltransferase (4PYIA, cyan); **Table S1.** Proteins with high sequence similarity from viruses from different Baltimore classes, with detailed results of BLASTp comparisons; **Table S2.** Pfam families comprising proteins from viruses with different genome types identified with HMMer [28]; **Table S3.** UniProt identifiers, domain boundaries and e-values of the proteins from Pfam families comprising proteins from viruses with different genome types identified with HMMer [28]; **Table S4.** Proteins with high structural similarity from viruses from different Baltimore classes, with detailed results of structure alignment.
